# Repetitive Transcranial Magnetic Stimulation for Alzheimer’s Disease Based on Apolipoprotein E Genotyping: Protocol for a Randomized Controlled Study

**DOI:** 10.3389/fnagi.2021.758765

**Published:** 2021-12-02

**Authors:** Naili Wei, Jian Chen

**Affiliations:** Department of Neurosurgery, The First Affiliated Hospital of Shantou University Medical College, Shantou, China

**Keywords:** repetitive transcranial magnetic stimulation (rTMS), Alzheimer’s disease (AD), APOE genotype, APOE4 carriers, randomized controlled Trail (RCT), preresults

## Abstract

To date, there is a shortage of effective treatment strategies for Alzheimer’s disease (AD), and although repetitive transcranial magnetic stimulation (rTMS) can improve AD cognitive function, there are obvious individual differences, which may be related to different apolipoprotein E (APOE) genotypes. As the risk and pathogenesis of AD varies greatly among different genotypes precise treatment strategies should be implemented depending upon genotype, which has not been proved by clinical studies. Apart from that, the published clinical studies are highly heterogeneous, and therefore, systematic and well-developed randomized controlled Trails (RCT) and demonstration of precise administration protocols are required. To verify this hypothesis, this project designed a RCT study, and randomly divided apoE4 carrier AD and non-carrier AD into high-frequency rTMS (HF-rTMS) or low-frequency rTMS (LF-rTMS) treatment groups. Specifically, 80 patients with AD, namely 48 APOE4 carriers and 32 non-APOE4 carriers will be included in the study. After that, based on different stimulation frequencies of rTMS, they will be divided into the HF-rTMS group and the LF-rTMS group, when patients with AD will be randomly assigned to different treatment groups. After AD patients are involved in the study, their memory, cognition, anxiety, depression and activities of daily living will be tested before and during 2 weeks of rTMS. Furthermore, peripheral blood will be collected before and after treatment to detect changes in pathological indexes via MSD platform (Meso Scale Discovery), while 32-channel EEG data will be also collected to detect and analyze changes in gamma oscillation. In addition, these patients will be followed up for 6 months and their neuropsychological scale was also evaluated every month. At present, our study has included 18 AD patients (10 APOE4 carriers; 8 non-carriers). Our study is still in progress. The grouping has not been unblinded. But the preliminary data demonstrated that non-carriers had better MoCA score improvement than APOE4 carriers. The results indicated that the two populations of AD patients should be treated differently. Thus, this project will provide direction for precision rTMS in AD and also promotes a shift in relevant treatment philosophy.

**Clinical Trial Registration:** [www.ClinicalTrials.gov], identifier [ChiCTR2100041625].

## Introduction

Alzheimer’s disease (AD) is a neurodegenerative disease characterized by memory loss and cognitive dysfunction, with a very high prevalence in the middle-aged and elderly population. In this context, hundreds of billions of dollars have been invested in related research and development worldwide, but unfortunately, there are still no drugs with proven efficacy. Moreover, patients may wander and lose their ability to live in the late stages, which is a heavy burden for society and families (Zhou et al., 2019). Therefore, investigating new treatments is urgently needed.

Repetitive transcranial magnetic stimulation (rTMS) is a safe, non-invasive, and inexpensive neuromodulation that can affect the synaptic plasticity of neurons and enhance brain function by adjusting parameters such as stimulation frequency and stimulation intensity (Bestmann, 2008). In recent years, some researchers have tried to adopt rTMS to treat Alzheimer’s disease and found that it has a better therapeutic effect (Dong et al., 2018; Chou et al., 2020). The current clinical treatment of Alzheimer’s disease with medications has unsatisfactory therapeutic effect. Zhang et al. (2020) reported a meta-analysis of pharmacological treatments showing that improvement of Mini-mental State Examination (MMSE) score standard mean difference (SMD) ranges from −0.29 to 0.49; Lu et al. (2020) also reported another analysis of anti- Aβ agents for mild to moderate Alzheimer’s disease suggesting no effect of anti- Aβ drugs vs. the placebo group (SMD of MMSE score improvement: −0.29, 95% CI −0.76–0.17; SMD of ADAScog score improvement: MD: 0.20, 95% CI: −0.40–0.81). In contrast, Chu et al. (2021) reported a promising result. The patients who received high-frequency TMS treatment had a significant improvement in MMSE score (1.65, 0.77–2.54) when compared with sham TMS. In addition, Dong et al. (2018) report also supported the viewpoint that high-frequency rTMS led to a significant improvement in cognition as measured by ADAS-cog (MD = −3.65, 95% CI −5.82 to −1.48). Although the results derived from different study samples, their subjects were AD patients. Although there are no studies designed to compare the differences in therapeutic effects between drugs and rTMS, these data point to the possibility that rTMS is superior to drug therapy of AD patients.

Unfortunately, the results of the published clinical studies on transcranial magnetic stimulation greatly differ, with different parameters and stimulation targets being used, and a wide variation in stimulation patterns (Dong et al., 2018; Chou et al., 2020). Specifically, there are obvious individual differences in patient response to transcranial magnetic stimulation (Dong et al., 2018; Chou et al., 2020). Therefore, the precise implementation of transcranial magnetic stimulation therapy is crucial.

How to precisely direct the rTMS strategy is remains unsolved. However, recent studies indicate that APOE gene-typing is a possible candidate. The apolipoprotein gene (APOE) that can be classified into E2, E3, and E4 subtypes based on the differences in the bases encoding the rs429358 and rs7412 loci but falls into six genotypes according to the double allele typing. The different genotypes play different roles in the pathogenesis of Alzheimer’s disease. The APOE ε2 allele proven to be the strongest genetic protective factor but the APOE ε4 allele was the strongest genetic risk factor for Alzheimer’s disease after multiple large scale genome-wide studies (Serrano-Pozo et al., 2021). Several researchers have called for the study and treatment of carriers and non-carriers as different phenotypic groups (Yamazaki et al., 2019; Serrano-Pozo et al., 2021). In addition, the other researchers found that APOE ε4 allele carriers had a different outcome of physical exercise treatment (Cancela-Carral et al., 2021). Therefore, we speculate that different APOE ε4 allele carriers have different outcomes of rTMS.

Its potential mechanism may be related to impairment of the function of GABAergic interneurons (Knoferle et al., 2014; Lenz et al., 2016; Wang et al., 2018). Many studies have suggested that the APOE4 allele can reduce the number and impair the function of GABA interneurons in the brain and increased cortical excitability (Knoferle et al., 2014; Lenz et al., 2016; Wang et al., 2018). This feature can also be confirmed by EEG results. High-frequency gamma oscillations are generally encoded by GABA interneurons in the brain (Traub et al., 2003; Colgin et al., 2009; Buzsaki and Wang, 2012; Gillespie et al., 2016). Inhibition of this neuronal activity can lead to a reduction in gamma oscillations, and the reduction of gamma oscillations in the brain of APOE4 carriers (Traub et al., 2003; Colgin et al., 2009; Buzsaki and Wang, 2012; Gillespie et al., 2016) is closely related to the symptoms of AD (Zhao et al., 2018; Etter et al., 2019; Najm et al., 2019). Transcranial magnetic stimulation can improve gamma oscillation power and amplitude, and improve cognitive function (Barr et al., 2009). These results suggest that the damage to gamma oscillation activities caused by the APOE4 allele may affect the treatment results. In summary, it is clear that the APOE genotype determines the number and function of GABAergic neurons in AD patients, and therefore may be related to the heterogeneity of transcranial magnetic stimulation effects. Considering this background, transcranial magnetic stimulation treatment protocols being implemented should vary with APOE genotypes.

Previous studies have tested the effect of both high-frequency rTMS (HF-rTMS) and low-frequency rTMS (LF-rTMS). Interestingly, it was found that both HF-rTMS of the left dorsolateral prefrontal cortex (L-DLPFC) and LF-rTMS of right dorsolateral prefrontal cortex (R-DLPFC) exhibited significant therapeutic efficacy (Chou et al., 2020). Additionally, Ahmed et al. (2012) reported that LF-rTMS of L-DLPFC also showed significant therapeutic efficacy for mild to moderate dementia. But there are no uniform guidelines or standards to guide the choice of treatment protocol. In terms of experience, most doctors will choose high frequency as the treatment scheme. The present study will adopt the left dorsolateral prefrontal cortex (L-DLPFC) as the intervention target.

We believe that different APOE4 carriers should be given different treatment schemes than non-APOE4 carriers because of the excitability of their neural networks is different. The APOE4 carriers have increased network excitability due to the impairment of GABAergic interneuron function (Najm et al., 2019). Compared with the APOE ε3 allele, APOE ε4 allele increases Ca^2+^ excitability due to lysosome dysregulation and impaired modulation of Ca^2+^ responses upon changes in extracellular lipids (Larramona-Arcas et al., 2020). Traditional theory suggests that high-frequency rTMS increases cortical excitability by inducing long-duration enhancement potentials, while low-frequency rTMS decreases cortical excitability by inducing long-duration inhibition potentials (Pell et al., 2011). GABAergic neuronal receptor activity is mainly associated with chloride channel opening (Traub et al., 2003), and high-frequency stimulation is more likely to induce long-term potentiation (LTP) (Pell et al., 2011). Hence, high-frequency treatment contributes to more gamma oscillatory activity and better therapeutic effects. However, for APOE4 carriers with more significant loss of GABAergic neurons (Knoferle et al., 2014; Najm et al., 2019), HF treatment may be less effective. In this case, low-frequency therapy may be more suitable for that kind of patients. Therefore, in this study, it is believed that different transcranial magnetic stimulation regimens should be selected for AD patients based on APOE genotypes, so as to obtain precise treatment strategies.

In summary, this paper aims to study the difference in the efficacy of different rTMS strategies in AD patients with different APOE genotypes, and analyze the changes of gamma oscillations.

## Objectives

The primary objective of the study is to analyze the different efficacies of rTMS in AD patients with different APOE genotypes, so as to provide the basis for the precise treatment of AD.

The secondary objective is to analyze the changes in gamma oscillations and pathological indicators in different APOE genotypes, thus being conducive to the precise treatment of AD.

## Methods

### The Recruitment and Inclusion of Alzheimer’s Disease Patients

The patients are recruited in two main ways: (1) Online recruitment: our recruitment program was announced on our hospital’s official website in February this year^1^. Then, all eligible Alzheimer’s disease patients could apply for rTMS treatment by filling in the online form.

(2) Hospital system: The hospital information retrieval system are also adopted to obtain information about patients with cognitive impairment and call to ask if they would like to participate in the recruitment program. Then, those who are interested in the program will enter into our patient recruitment diagnostic process.

**FIGURE 1:**
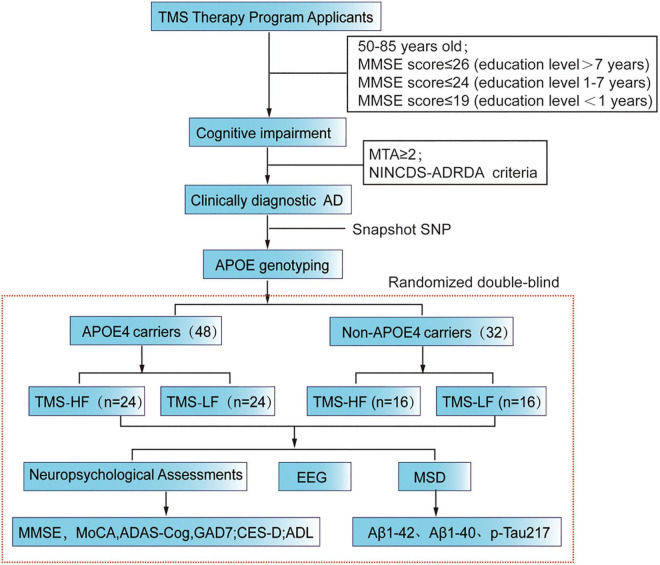
Flow chart of the present RCT study. MMSE, Mini Mental State Examination; rTMS, repetitive transcranial magnetic stimulation; MoCA, Montreal Cognitive Assessment Scale; ADAScog, Assessment Scale-cognitive subscale; Center for Epidemiologic Studies Depression Scale (CES-D); Activity of Daily Living (ADL); MSD, Meso Scale Discovery.

These candidates applying for rTMS treatment will undergo neuropsychological assessments in the outpatient clinic of our hospital. Then, those with cognitive impairment are recommended for MRI scanning so as to assess the extent of hippocampal atrophy, while those with an MTA score ≥ 2 are the potential AD patients, which are diagnosed by the neuroimaging specialist (Figure 1). In addition to that, the data of these patients will be further evaluated by two neurologists. Furthermore, the eligible applicants for a clinical diagnosis of Alzheimer’s disease should meet our inclusion criteria and exclusion criteria. Beyond that, they should sign a subject informed consent form with our research team. Here, it should be mentioned that before rTMS treatment, patient blood samples were collected for APOE genotyping (Table 1).

**TABLE 1:** APOE genotyping is determined by promoter polymorphisms of two single nucleotides (rs429358 and rs rs7412).

APOE genotypes	S1:rs429358	S2:rs7412
E2/E2	T/T	T/T
E3/E3	T/T	C/C
E4/E4	C/C	C/C
E2/E3	T/T	T/C
E2/E4	T/C	T/C
E3/E4	T/C	C/C

*T or C represents the first DNA base of the nucleotides.*

The inclusion and exclusion criteria are as follows:

**Inclusion criteria**

(1) The age ranges from 50 to 85 years old.
(2) Subjective perception of memory loss more than 1 year; MMSE ≤ 26 (level of education more than 7 years: MMSE ≤ 26; level of education 1–7years: MMSE ≤ 24; level of education less than 1 year: MMSE ≤ 19).
(3) MRI exhibits medial temporal atrophy (hippocampus, entorhinal cortex, amygdala); MTA visual evaluation scale ≥ 2.

**Exclusion criteria**

(1) There was a history of severe brain lesions or brain surgery, such as brain tumor, hydrocephalus and intracranial hematoma.
(2) The patients have received TMS treatment, transcortical electrical stimulation, deep brain electrical stimulation, or vagus nerve electrical stimulation prior to application.
(3) There are serious complications, such as frequent seizures, brain tumor surgery, intracranial infection, serious heart disease, lung disease, mania, and severe mental disorders.
(4) The patient had an implanted pacemaker, defibrillator, cochlear implant, other nerve stimulator, or steel plate, and could not accept TMS therapy or MRI scanning.
(5) The patient suffers frontotemporal dementia, vascular dementia, Lewy body dementia, or other diseases.
(6) White matter lesions with a Fazekas score > 3 and cerebral infarction in medial temporal lobes were excluded.
(7) Others referred to the NINCDS-ADRDA exclusion criteria (revised—2007) (Dubois et al., 2007).

**Exit criteria**

(1) Subjects voluntarily withdraw from the study for personal reasons.
(2) Subjects are unable to continue to participate in the study due to a serious medical condition, such as a cardiovascular accident, or those that would have a significant impact on the study results.
(3) Subjects developed serious treatment-related complications, are unable to tolerate the treatment dose or could not complete the associated assay.

### Apolipoprotein E Genotyping

DNA was extracted from the patients’ peripheral blood. Then, genotyping was performed by snapshot SNP typing. In addition, APOE alleles and genotypes were determined by sequencing rs429358 and rs7412 at exon 4 of the APOE gene. Furthermore, APOE status was defined by the possessing one or more copies of E2, E3 and E4, whereas APOE ε4 positive status was confirmed as the ε4/ε4; ε3/ε4 or ε2/ε4 (Risacher et al., 2015). In the experimental steps, 2 ml anticoagulant peripheral venous blood was taken to extract leukocytes, genomic DNA was extracted from blood samples by silicon matrix adsorption column method for PCR amplification of target genes, and then the PCR products were purified. After snapshot extension reaction, sequencing and analysis were carried out. The primer sequences used for genotyping were:rs429358-F: AATC GGAACTGGAGGAACAAC; rs429358-R: GATGGCGCTGAG GCCGCGCTC; rs7412-F: AATCGGAACTGGAGGAACAAC; rs7412-R: GATGGCGCTGAGGCCGCGCTC.

### Randomization and Allocation

#### Stratified Randomized Groups

In this study, 48 APOE4 carrier AD patients (APOE4-AD) and 32 non-APOE4 carrier AD patients (Non-APOE4-AD) will be recruited. Then, they will be assigned to the HF-rTMS group and the LF-rTMS group based on the stratified randomization method: 40 for the HF-rTMS group and 40 for the LF-rTMS group, while in subgroups, 24 patients will be assigned to in the APOE4-HF group, 24 patients will be assigned to the APOE4-LF group, 16 patients will be assigned to non-APOE4-HF group, and 16 will be assigned to the non-APOE4-LF group. The specific process is as follows: Our team members consist of quality control personnel, TMS therapy physicians, neuropsychological assessors and laboratory staff. Specifically, quality control personnel are responsible for quality control of study data and grouping randomization; TMS therapy physicians perform TMS treatment based on information from quality control personnel; neuropsychological assessors conduct neuropsychological assessments before, during and after treatment and follow-up; laboratory staff are in charge of blood sample processing and testing. In addition, the grouping randomization information will not be released by quality control personnel until the primary endpoint of the study has been met for all program participants at follow-up (Table 1).

The postadmission randomization process are performed by the quality control staff. Specifically, the admission sequence of APOE4 carriers and non-carriers was 1–48 and 1–32, respectively, and the admission numbers of carriers and non-carriers were randomized using Excel sheet randomization to assign them within the HF-rTMS and LF-rTMS groups, respectively. When the follow-up of all subjects reached the clinical trial endpoint, the project quality control staff and the project leader verified the grouping information for unblinding and summarized the project data for statistical analysis.

### Repetitive Transcranial Magnetic Stimulation Therapy

HF-rTMS group: The rTMS therapy are performed by a transcranial magnetic stimulator (XY-K-JLC-D, Xiangyu Medical, China).

#### TMS Target: Left Dorsolateral Prefrontal Cortex (L-DLPFC)

The patients accepted 14 consecutive days of TMS therapy (twice a day), with each treatment lasting for 30 min (110% of motor threshold, 20 Hz, 1 s, 29 s interval, 1200 pulses per treatment, 33,600 total sessions).

LF-rTMS group: The rTMS therapy was performed by transcranial magnetic stimulator (XY-K-JLC-D, Xiangyu Medical, China).

#### TMS Target: Left Dorsolateral Prefrontal Cortex (L-DLPFC)

The patients accepted 14 consecutive days of TMS therapy (twice a day) with each treatment lasting for 30 min (110% of motor threshold, 1 Hz, he stimulation time 40 s, 20 s interval, 1200 pulses per treatment, 33,600 total sessions).

### Localization of Transcranial Magnetic Stimulation Target

First, the skull surface position of the left precentral gyrus are located. Specifically, the midpoint of bilateral cerebral hemispheres will be confirmed by that of the patient’s bilateral tragus above the skull. Then, the location of the superior sagittal sinus was identified by connecting the nasal root position, the midpoint of bilateral cerebral hemispheres and the external occipital protuberance. It should be mentioned that the midpoint of this line is that of the superior sagittal sinus, which corresponds to the location of the central sulcus. Then, the skull surface position of the hand representation area in the central anterior gyrus was measured by 5 cm left siding from this midpoint in a direction perpendicular to the sagittal line. Furthermore, the left dorsolateral prefrontal region, the target area for rTMS, was located at 20% of the sagittal sinus length from this point in parallel to the sagittal line in a forward translation.

### Study Process

After AD patients are included in the study, their neuropsychological scales, including cognition, anxiety, depression, activities of daily living and other scales will be assessed. Then, they will be assigned to HF-rTMS or LF-rTMS group and accept due treatment for 2 weeks. During the treatment, the neuropsychological scales will be assessed every week. In addition, blood test results and 32-channel EEG data will be collected before and after treatment, while a 6-month follow-up will be carried out after treatment (Figure 1).

### Neuropsychological Assessments

The Mini Mental State Examination (MMSE), Montreal Cognitive Assessment Scale (MoCA) and Alzheimer’s Disease Rating Scale cognitive subscales (ADAS-cog) will be used to assess cognitive function, while the Generalized and Center for Epidemiologic Studies Depression Scale (CES-D) will be adopted to assess the mood of all the participants. In addition, daily activity is measured using the Activity of Daily Living (ADL) scale. All scales are tested with common forms and unified guidelines. The evaluators all have received standardized training and they will communicate with the subjects in local language.

### EEG Acquisition and Calculation and Analysis of EEG Metrics

EEG data will be collected via a high-density 32-channel EGI system (g.tec) with a sampling rate of 500 Hz and a mastoidea reference. During the recording, patients are instructed to stay awake, relax, and close their eyes for 10 min. Then, a bandpass filter (from 0.5 to 200 Hz) and a notch filter at 50 Hz and 100 Hz were applied. In addition, EEG will be collected under the condition of eyes closed, followed by preprocessing via MATLAB software, such as baseline adjustment, filtering, depression filtering, and referring. Finally, the changes in EEG power and spectra in different frequency bands before and after treatment of different genotypes were analyzed and compared.

EEG metric data will be processed and analyzed by MATLAB 2017. Beyond that, power spectral density (PSD), median spectral frequency (MSF) and spectral entropy measure dynamics of brain signals at a single electrode site are based on spectral frequency content.

For the main analysis, 10 EEG metrics: PSD in delta (1–4 Hz), theta (4–8 Hz), alpha1 (8–10 Hz), alpha2 (10–13 Hz), beta 1(13–20 Hz), beta 1(20–30 Hz), gamma1 (30–48 Hz), and gamma1 (48–100Hz) will be calculated, and then averaged across all epochs (60 s recording).

### Outcomes

The primary end point is the quantitative index, namely MMSE, whereas the secondary outcome measures are MoCA, ADAS-cog, GAD-7, CES-D, and ADL.

### Sample Size Calculation

The sample size of this project was calculated by Pass software on the basis of previous literature. In this study, the MMSE scores of the HF-rTMS group and the LF-rTMS group were 10.4 ± 1.7 and 10.2 ± 1.8, respectively, before treatment. After 2 weeks of rTMS treatment, their MMSE scores reached 11.2 ± 1.9 and 8.8 ± 2.3, respectively (Ahmed et al., 2012), when the target effect size had 80% power (β = 0.10) and a type I error was 5% (α = 0.05). In addition, the group sample size of 13 and 12 achieved 81% power for detecting a difference of 2.4 between the null hypothesis and that the mean of both groups is 11.2 and the alternative hypothesis that the mean of Group 2 is 8.8 with known group standard deviations of 1.9 and 2.3 and a significance level (alpha) of 0.050 using a two-sided two-sample *t*-test, while the sample size of each subgroup is at least 13. Considering the loss of follow-up, falling off and sample data being eliminated due to data quality, the sample size should be increased by 20%. Thus, the final sample size of each subgroup are at least 16. Furthermore, the ratio of APOE4 carriers to non-APOE4 carriers are set to 3:2 according to the comparison of population samples and our previous study. Other than that, in this study, 80 participants, with 48 APOE4 carriers and 32 non-APOE4 carriers will be recruited.

### Quality Control and Quality Assurance

Two neuroscientists, a neuroimaging specialist and an expert in geriatrics, worked together to examine the participants and provide a diagnosis for each participant. In addition, training was provided for all researchers, while all items involved in the test, such as blood samples, EEG data processing and neuropsychological assessments were monitored by the quality control committee who randomly selected data samples to send to a third party for retesting or re-evaluation. Data entry was performed by EpiData Entry software and was also monitored by the quality control committee who further randomly selected the input information and paper information. To control the impacts of clinical treatment, we strictly restricted medication. Oral medication before and during hospitalization as well as during follow-up remained unchanged except for sudden diseases, such as cardiovascular and cerebrovascular accidents and aggravation of primary diseases. If there are errors, the sampling proportion will be expanded, while if the input error proportion is high, a second person needs to check and correct the input data. Moreover, all data were monitored and reviewed by the principal investigator or research coordinators.

### Biomarker Test of Blood Samples

Aβ1-42, Aβ1-40, p-tau217 and other indicators of the plasma were detected by liquid phase flow cytometry (using the MSD platform) before and after treatment to evaluate the outcome of pathological indicators. Specifically, blood samples were collected at 7:00 a.m. before and after treatment, and plasma samples were obtained after centrifugation for MSD detection.

### Statistical Methods

#### Statistical Analysis Methods

(1) Descriptive analysis: the counting data was presented as the mean ± standard error; (2) the counting data was compared between groups by performing the continuous correction χ^2^-test, when the theoretical frequency of more than 25% cells was less than 5. The Fisher exact probability method was adopted; the independent sample *t*-test was conducted for comparison between groups of normally distributed measures; repeated-measures ANOVA was employed to analyze repeated data between groups in different time. For non-normally distributed measures, the Wilcoxon rank sum (WRS) test was used for comparisons between groups.

#### Statistical Hypothesis

The primary endpoint of this study was the end of treatment and completion of the 3-month follow-up for all subjects, and its indicator was the MMSE score. Apart from that, the study hypothesis is to demonstrate the superiority of HF-rTMS over LF-rTMS in transcranial magnetic stimulation protocols for patients with Alzheimer’s disease. In terms of subgroup analysis, the HF treatment group was preferentially compared with the low-frequency treatment group of non-APOE4 carriers; if the statistical result of *P*-value is less than 0.05, then the comparison between the HF treatment group and the low-frequency treatment group of APOE4 carriers is made in order. If the statistical result of *P*-value is less than 0.0125, then the comparison among the four subgroups is considered significant.

### Ethics and Dissemination

This study protocol was been approved by the Clinical Research Ethics Committee of the First Affiliated Hospital of Shantou University Medical College (approval number: 2020-115-XZ2). Apart from that, our team will inform all participants individually of detailed information about our research, while informed consent will be obtained from all participants and/or their legal representatives. In addition, participants will be allowed to withdraw from the study any time, but the reason will be recorded. Furthermore, the results of the study will be reported in peer-reviewed journals and presented at national or international conferences on neuromodulation.

### Results in Progress

Our study is still in progress. Our current data are the preliminary data of the RCT study, and the grouping has not been unblinded. We analyzed the demographic informatics of APOE4 carriers and non-carriers and the changes in cognitive function scores before and after 2 weeks of rTMS treatment. More detailed information was displayed in Supplementary Table 1.

**FIGURE 2:**
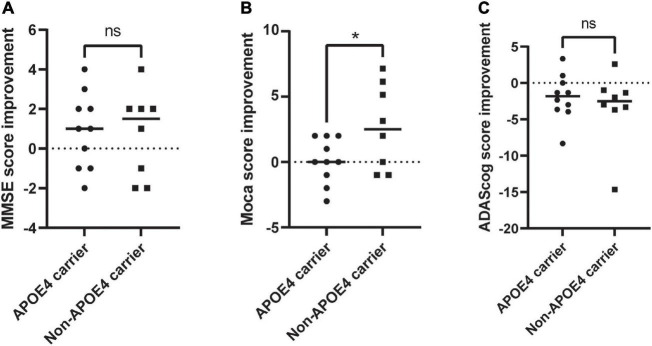
Evaluation of the changes in neuropsychological scale of APOE4 carries and noncarriers pre-rTMS and post-rTMS. **(A)** MMSE improvement; **(B)** MoCA improvement; **(C)** ADAScog improvement. rTMS, repetitive transcranial magnetic stimulation; MoCA, Montreal Cognitive Assessment Scale; ADAScog, Assessment Scale-cognitive subscale. *Indicates of a *P*-value < 0.05.

To date, 18 patients with clinically diagnosed Alzheimer’s disease have been included and have received rTMS treatment for 2 weeks. Among them, ten were APOE4 carriers; eight were non-carriers, and there were no significant differences in age, education or gender (Table 2). They were randomly assigned to the high-frequency treatment group or the low-frequency treatment group. Because the study was not blinded, we could not know which subgroup they were assigned to. We could only analyze the overall treatment effect. According to our preliminary results, there was no significant difference in MMSE score, MoCA score except ADAScog (Supplementary Table 1) score between the two groups before and after treatment. When comparing the improvement rate of the two groups, there was a significant difference in the improvement of MoCA score between the two groups. For non- carriers, the MoCA score but not the MMSE or ADAScog score improved by 2.65 ± 3.15, while for carriers, it improved by 0.1 ± 1.7 (*t* = 2.164, *p* = 0.046, Figures 2A–C).

**TABLE 2:** Characteristics of the study cohort.

	APOE4 carriers (*n* = 10)	Non-APOE4 carriers (*n* = 8)	*p*-value
Sex F/M	6/4	5/3	0.914
Age, years	70.37.45	73.756.27	0.312
Education level, years	7.72.98	6.135.37	0.44
MMSE score before rTMS	12.66.77	15.888.54	0.377
MoCA score before rTMS	7.94.28	9.255.50	0.566
ADAScog score before rTMS	39.1316.49	34.8719.03	0.618
MMSE score after rTMS	13.56.22	16.638.73	0.388
MoCA score after rTMS	84.47	11.887.72	0.202
ADAScog score after rTMS	37.1616.39	31.5721.21	0.536

*n, number; F, female; M, male; MMSE, Mini Mental State Examination; rTMS, repetitive transcranial magnetic stimulation; MoCA, Montreal Cognitive Assessment Scale; ADAScog, Assessment Scale-cognitive subscale.*

## Discussion

Transcranial magnetic Stimulation (TMS) is a magnetic stimulation technique that uses a transient magnetic field to generate induced currents in the cerebral cortex, thus altering the membrane potential of cortical neurons (Bestmann, 2008). In addition, it is also a painless, non-invasive, safe and reliable tool for non-invasive physiotherapy and research on the central and peripheral nervous systems (Pell et al., 2011). Since the invention of transcranial magnetic stimulation by Anthony Barker from the University of Sheffield in 1985, this technique has been rapidly applied to neuroscience research and clinical practice (Pell et al., 2011). Specifically, in the past 20 years, the number of articles published on TMS has geometrically increased. At present, TMS has been widely used in neuroscience, neurology, psychiatry, rehabilitation medicine, medical psychology and other fields.

At present, there is no effective drug for Alzheimer’s disease (Lu et al., 2020; Zhang et al., 2020). Doctors or researchers in many fields are trying variable treatment methods. Transcranial magnetic stimulation is a form of non-invasive nerve regulation, which has very good prospects in the treatment of AD (Dong et al., 2018; Chou et al., 2020; Chu et al., 2021). Several meta-analyses demonstrated the superiority of transcranial magnetic stimulation in the treatment of AD (Dong et al., 2018; Chou et al., 2020; Chu et al., 2021). However, at present, the number of RCTs remain relatively few. Different researchers in previous studies adopted different stimulation targets, intensities, and frequencies, resulting in great differences among these studies. At present, the rTMS protocol continues to rely on empirical selection, and there is no unified understanding of the reference basis. More importantly, according to many published research data, there are great individual differences among different patients in the same research project. This individualized difference also puts forward the demand for accurate rTMS schemes.

Our project hypothesis holds that the treatment scheme can be selected according to the APOE genotyping of Alzheimer’s disease. Although our study has not been unblinding, the preliminary results showed the difference in the treatment effect between APOE4 carriers and non-carriers. Thus, it is very necessary to treat APOE4 carriers and non-carriers differently in the treatment scheme. Precise neuromodulation is also the future trend. Furthermore, the current research of our project team is to guide the precise treatment of Alzheimer’s disease through different gene phenotypes, thus providing guidance for precise and non-invasive neural regulation of AD, and promoting the transformation of transcranial magnetic stimulation in AD treatment.

## Ethics Statement

The studies involving human participants were reviewed and approved by the Clinical Research Ethics Committee of the First Affiliated Hospital of Shantou University Medical College (approval number: 2020-115-XZ2). The patients/participants provided their written informed consent to participate in this study. Written informed consent was obtained from the individual(s) for the publication of any potentially identifiable images or data included in this article.

## Author Contributions

NW: conception, supervision, and design of this article. JC: manuscript editing and research fund. Both authors in the article have approved the submitted version.

## Conflict of Interest

The authors declare that the research was conducted in the absence of any commercial or financial relationships that could be construed as a potential conflict of interest.

## Publisher’s Note

All claims expressed in this article are solely those of the authors and do not necessarily represent those of their affiliated organizations, or those of the publisher, the editors and the reviewers. Any product that may be evaluated in this article, or claim that may be made by its manufacturer, is not guaranteed or endorsed by the publisher.

## Footnotes

1
http://www.stuh.com.cn/index.php/home/view?id=6175
