# Supplementary material for: Repetitive Transcranial Magnetic Stimulation for Alzheimer’s Disease Based on Apolipoprotein E Genotyping: Protocol for a Randomized Controlled Study
Source: Front Aging Neurosci. 2021 Dec 2;13:758765. doi: 10.3389/fnagi.2021.758765 (PMC8675398; doi:10.3389/fnagi.2021.758765)
Supplement: Supplementary file 1 [file Table_1.DOC]

**Supplementary materials**

1. Table-1 Information of Pre-rTMS and Post-rTMS in AD patients

| Patients | SEX | Age  (years) | Education  Level  (years) | APOE  genotypes | Pre-rTMS evaluation | | | Post-rTMS evaluation | | |  |
| --- | --- | --- | --- | --- | --- | --- | --- | --- | --- | --- | --- |
|  |  |  |  |  | MMSE | MoCA | ADAScog | MMSE | MoCA | ADAScog^*^ |  |
| 1 | F | 64 | 12 | E3/E3 | 25 | 17 | 11.3 | 27 | 22 | 9.33 |  |
| 2 | F | 72 | 3 | E3/E3 | 22 | 12 | 27 | 24 | 19 | 12.334 |  |
| 3 | M | 79 | 16 | E2/E3 | 4 | 3 | 64 | 8 | 6 | 66.6 |  |
| 4 | F | 77 | 7 | E3/E3 | 26 | 14 | 22 | 27 | 20 | 21 |  |
| 5 | M | 82 | 3 | E2/E3 | 11 | 6 | 28.67 | 13 | 8 | 25 |  |
| 6 | M | 78 | 5 | E2/E3 | 10 | 7 | 45.33 | 9 | 6 | 44 |  |
| 7 | F | 67 | 0 | E3/E3 | 8 | 2 | 59 | 6 | 1 | 55.67 |  |
| 8 | F | 71 | 3 | E3/E3 | 21 | 13 | 21.667 | 19 | 13 | 18.667 |  |
| 9 | F | 58 | 9 | E3/E4 | 2 | 3 | 63 | 6 | 3 | 64 |  |
| 10 | M | 77 | 12 | E3/E4 | 11 | 9 | 50.66 | 12 | 7 | 48.33 |  |
| 11 | F | 72 | 6 | E4/E4 | 20 | 13 | 32.66 | 18 | 10 | 32.667 |  |
| 12 | F | 67 | 3 | E4/E4 | 10 | 5 | 38.33 | 9 | 4 | 41.667 |  |
| 13 | F | 75 | 12 | E4/E4 | 8 | 3 | 53.3 | 9 | 5 | 49.66 |  |
| 14 | M | 78 | 5 | E3/E4 | 21 | 14 | 16.33 | 21 | 15 | 15 |  |
| 15 | M | 73 | 6 | E3/E4 | 22 | 11 | 13.66 | 24 | 13 | 12.33 |  |
| 16 | F | 58 | 6 | E3/E4 | 9 | 3 | 46.33 | 8 | 3 | 38 |  |
| 17 | M | 68 | 9 | E3/E4 | 7 | 7 | 50.33 | 10 | 7 | 46.372 |  |
| 18 | F | 77 | 9 | E3/E4 | 16 | 11 | 26.66 | 18 | 13 | 23.67 |  |

F, female; M, male; MMSE, mini mental state examination; rTMS, repetitive transcranial magnetic stimulation; MoCA, Montreal cognitive assessment scale; ADAScog, Assessment Scale-cognitive subscale. * indicates the significant difference of ADAScog changes after treatment using paired samples t-test (t=2.70; p=0.015)
